# Non-typhoidal *Salmonella* serotypes, antimicrobial resistance and co-infection with parasites among patients with diarrhea and other gastrointestinal complaints in Addis Ababa, Ethiopia

**DOI:** 10.1186/s12879-015-1235-y

**Published:** 2015-11-04

**Authors:** Tadesse Eguale, Wondwossen A. Gebreyes, Daniel Asrat, Haile Alemayehu, John S. Gunn, Ephrem Engidawork

**Affiliations:** Aklilu Lemma Institute of Pathobiology, Addis Ababa University, P.O. Box 1176, Addis Ababa, Ethiopia; Department of Veterinary Preventive Medicine, The Ohio State University, 1920 Coffey Rd., Columbus, Ohio 43210 USA; Department of Microbiology, Immunology & Parasitology, School of Medicine, College of Health Sciences, Addis Ababa University, Churchill Avenue, P.O. Box 9086, Addis Ababa, Ethiopia; Department of Microbial Infection and Immunity, Center for Microbial Interface Biology, The Ohio State University, Biomedical Research Tower, 460 West 12th, Columbus, OH 43210-1214 USA; Department of Pharmacology and Clinical Pharmacy, School of Pharmacy, College of Health Sciences, Addis Ababa University, Churchill Avenue, P.O. Box 1176, Addis Ababa, Ethiopia

**Keywords:** Non-typhoidal *Salmonella*, Antimicrobial resistance, Prevalence, Serotype

## Abstract

**Background:**

Non-typhoidal *Salmonella* (NTS) is an important public health problem worldwide. Consumption of animal-derived food products and direct and/or indirect contact with animals are the major routes of acquiring infection with NTS. Published information, particularly on the serotype distribution of NTS among human patients with gastroenteritis and associated risk factors, is scarce in Ethiopia. This study investigated the prevalence, risk factors, serotype distribution and antimicrobial susceptibility of *Salmonella* species among diarrheic out-patients attending health centers in Addis Ababa and patients with various gastrointestinal complaints at Tikur Anbessa Specialized Hospital (TASH).

**Methods:**

Stool samples were cultured for *Salmonella* species according to the WHO Global Foodborne Infections Network laboratory protocol. *Salmonella* serotyping was conducted using slide agglutination and microplate agglutination techniques. Antibiotic susceptibility testing was performed using the disk diffusion method according to Clinical and Laboratory Standards Institute guidelines.

**Results:**

A total of 59 (6.2 %) stool samples, out of 957 were culture positive for *Salmonella* species. Fifty-five (7.2 %) of 765 diarrheic patients from health centers and 4 (2.1 %) of 192 patients from TASH were culture positive for *Salmonella* species. Multivariable logistic regression analysis after adjusting for all other variables revealed statistically significant association of *Salmonella* infection with consumption of raw vegetables (OR = 1.91, 95 % CI = 1.29–2.83, χ^2^ = 4.74, *p =* 0.025) and symptom of watery diarrhea (OR = 3.3, 95 % CI = 1.23–8.88, χ^2^ = 10.54, *p =* 0.005). Eleven serotypes were detected, and the most prominent were *S.* Typhimurium (37.3 %), *S*. Virchow (34 %), and *S.* Kottbus (10.2 %). Other serotypes were *S.* Miami, *S.* Kentucky, *S*. Newport, *S*. Enteritidis, *S*. Braenderup, *S*. Saintpaul, *S*. Concord and *S.* V:ROUGH-O. Resistance to three or more antimicrobials was detected in 27 (40.3 %) of the isolates. Resistance to five or more antimicrobials was detected in 17 (25.4 %). Resistance to individual antimicrobials was found at varying proportions: streptomycin (50; 74.6 %), nitrofurantoin (27; 40.3 %), sulfisoxazole (26; 38.8 %), kanamycin (23; 34.3 %), cephalothin (12; 17.9 %), and ampicillin (11; 16.4 %) respectively. Two *S*. Kentucky, one *S*. Typhimurium and one *S.* Concord isolates were multi-drug resistant to more than 10 antimicrobials.

**Conclusions:**

The study demonstrated significant association of *Salmonella* infection with consumption of raw vegetables. There was no significant association of *Salmonella* infection with co-occurring parasites. The study also showed the dominance of *S.* Typhimurium and *S*. Virchow in primary health care units. Overall, prevalence of MDR was low compared to previous studies. Although their proportion was low, *S.* Kentucky and *S.* Concord demonstrated wider spectrum of MDR. Continuous monitoring of circulating serotypes, antimicrobial resistance profile and characterization on molecular resistance determinants is essential for proper treatment of patients and for identifying potential environmental origins of antimicrobial resistance.

**Electronic supplementary material:**

The online version of this article (doi:10.1186/s12879-015-1235-y) contains supplementary material, which is available to authorized users.

## Background

Non-typhoidal *Salmonella* (NTS) is an important public health problem worldwide. Globally, NTS is estimated to be responsible for 93.8 million cases of gastroenteritis and 155,000 deaths annually [1]. Unlike typhoid fever, which is mainly limited to developing countries, NTS occurs worldwide. Despite global morbidity, mortality due to NTS infection primarily occurs in the developing world and is related to co-morbidities [2]. Consumption of animal-derived food products and direct and/or indirect contact with animals are the major routes of acquiring infection with NTS [3–6]. NTS can also be transmitted from person to person or from contact with pets such as cats, dogs, rodents, reptiles, or amphibians [3, 4, 7, 8]. Several recent outbreaks have also been associated with consumption of contaminated plant products such as sprouts, tomatoes, fruits, peanuts, and spinach [9–11].

NTS usually causes self-limiting gastroenteritis characterized by diarrhea, abdominal pain and vomiting in people of all ages [12]. However, in children, the elderly and immunocompromised patients, severe invasive disease with complicated extra-intestinal illness, bacteremia and meningitis can be observed [7, 13]. Generally, antibiotic treatment is not necessary for NTS gastroenteritis unless it is invasive salmonellosis or it affects immunocompromised patients [14, 15]. Chemotherapy is believed to prolong the duration of shedding of bacteria and contribute to the development of antimicrobial resistance [16].

The relative importance of major etiologic agents responsible for diarrhea is not well established in Ethiopia. A few studies conducted in different parts of the country have shown that *Campylobacter*, *Salmonella, Shigella* and rotavirus are some of the major bacterial and viral pathogens isolated from stool of diarrheic patients, most of them children under 5 years of age [17–20].

Globally, the incidence of *Salmonella* infection associated with multi-drug resistance (MDR) has increased in the last few decades [21–23]. In Ethiopia, although there are a few studies on prevalence of *Salmonella* and antimicrobial susceptibility in humans, animals, and food of animal origin, there is no integrated surveillance and monitoring to establish the major serotypes responsible for non-typhoidal salmonellosis in humans and the associated risk factors. Moreover, most of the studies conducted in humans involved pediatric diarrheic patients and the isolates recovered from these patients were not sereotyped [24, 25]. Those that conducted serotyping indicated that *Salmonella enterica* subspecies enterica serovar Concord (*S.* Concord) and *Salmonella enterica* subspecies enterica serovar Typhimurium (*S.* Typhimurium) were the dominant NTS serotypes isolated from patients with diarrheal illness in Ethiopia [20, 26].

Information on *Salmonella* serotypes circulating in a given geographic area and their antimicrobial susceptibility is essential for designing appropriate intervention strategy since the outcome of infection varies with serotypes involved and their antimicrobial resistance profile. The objectives of the current study were therefore to investigate the prevalence, serotype distribution and antimicrobial resistance pattern of NTS species among diarrheic patients in 10 primary health centers in Addis Ababa. In addition, patients with gastrointestinal complaints who submitted stool samples to Tikur Anbessa Specialized Hospital (TASH) for bacterial culturing, diagnosis of gastrointestinal parasites and detection of *Helicobacter pylori* antigen were also included in the study. TASH is national referral hospital giving services mainly to patients from all sub-cities in Addis Ababa and those from outside Addis Ababa. Various patient related variables such as consumption of raw meat, milk and vegetables; patients demographic data; stool consistency; and the occurrence of other pathogens were recorded to analyze for possible association with *Salmonella* isolation.

## Methods

### Study design and area

A cross-sectional study was conducted in Addis Ababa, Ethiopia, from May 2013 to January 2014. Addis Ababa is organized under 10 sub-cities and there are 36 government-owned primary health centers in the city. Ten representative government-owned health centers were randomly selected, one from each sub-city. In addition, patients with various gastrointestinal complaints at TASH were also included.

### Sample size determination and study subjects

Sample size (*n =* 576) was calculated based on the previous baseline study in Addis Ababa, with a prevalence of 6.4 % [25] using the formula (Z0/2)/∆)2p(1-p) at 95 % CI, margin of error of 2 %. However, to increase the number of isolates, a total of 765 patients from health centers were included. From each health center, a minimum of 71 and a maximum of 82 diarrheic patients of any age referred for laboratory diagnosis based on stool examination were recruited (*n =* 765). Diarrhea was defined as the passage of three or more loose or liquid stools per day [27]. In addition, 192 patients who presented with various gastrointestinal complaints (diarrhea, abdominal pain and gastritis) and submitted stool samples to the parasitology and microbiology laboratory at TASH were also included. Only 98 (51 %) of the 192 stool samples from TASH were diarrheic.

### Patients’ history, demographic and laboratory data

Information on various potential risk factors was collected including patients’ history such as consumption of raw meat, milk and vegetables during the last 2 weeks by interviewing the patients during sample collection from health centers. All demographic, clinical and laboratory data obtained from study participants at health facilities such as stool consistency and laboratory examination results of stool specimens for other pathogens were recorded. The stool consistency was determined in the health center and hospital laboratories immediately after samples were received according to the Bristol stool consistency scale (type 5, 6 and 7) defined as loose, mucoid and watery, respectively [28]. Analysis for association of *Salmonella* infection status and various risk factors was conducted only for patients from health centers.

### Sample collection, handling and transportation

Stool samples were collected from each study participants in clean screw capped plastic containers and transported to the microbiology laboratory of Aklilu Lemma Institute of Pathobiology (ALIPB) in ice box within 3–4 h of collection for culture and identification of *Salmonella* species. Sampling was first conducted from the 10 health centers sequentially and finally from TASH.

### Microscopic examination of stool specimens

Direct microscopic stool examination was performed for detection of ova and parasites by laboratory technicians in health centers and hospital laboratories where the samples were collected immediately prior to transportation to ALIPB and the laboratory result was recorded.

### Culture and identification of *Salmonella* species

Isolation and identification of *Salmonella* species was conducted according to World Health Organization (WHO) Global Foodborne Infections Network laboratory protocol [29]. Briefly, 5 g of feces was suspended in 45 ml of buffered peptone water and incubated for 24 h at 37 °C. One hundred μl of this suspension was transferred to 10 ml of Rappaport-Vassiliadis enrichment Broth (RVB), (Oxoid, USA) and incubated for 24 h at 37 °C. One ml of suspension was also transferred to 10 ml of Tetrathionate broth (Oxoid, USA) and incubated for 24 h at 42 °C. The sample from these two broths was streaked on to Xylose Lysine tergitol 4 (XLT-4) selective media and the plates were incubated at 37 °C for 24 h. Presumptive *Salmonella* colonies were then further investigated biochemically using Triple Sugar Iron (TSI) agar, Urea, Citrate and Lysine Iron Agar (LIA) slants. Those colonies with typical *Salmonella* biochemical properties were then further confirmed by genus specific PCR [30]. *Salmonella* recovered from both RVB and TTB of a single patient were first considered as different strains until the isolates were tested for antimicrobial susceptibility. When differences in antimicrobial susceptibility were observed, both isolates were considered as different strains. On the other hand, if the isolates showed similar susceptibility pattern, only one isolate was considered for further analysis.

### *Salmonella* serotyping and phage typing

*Salmonella* isolates were serotyped and phage-typed at the Public Health Agency of Canada, World Organization for Animal Health (OIÉ) Reference Laboratory for Salmonellosis, Guelph, Ontario, Canada. Briefly, the somatic (O) antigens were determined by slide agglutination tests [31] and flagellar antigens were determined using a microplate agglutination technique [32]. The antigenic formulae of Grimont [33] were used to identify and assign the serotypes of the isolates. Phage typing of *S.*Typhimurium isolates was performed by the methods developed by Callow [34] and extended by Anderson et al. [35] with reference phages obtained from the Public Health England, Gastrointestinal Bacteria Reference Unit, Colindale, England and the Public Health Agency of Canada, National Laboratory for Enteric Pathogens, Winnipeg, Canada. *Salmonella* isolates that reacted with the phages but did not conform to any recognized phage type were designated atypical (AT).

### Antimicrobial susceptibility testing

Susceptibility of the isolates to a panel of 18 antimicrobials was determined using the Kirby-Bauer disk diffusion method according to the guidelines of the Clinical and Laboratory Standards Institute [36]. The following antimicrobials (Sensi-Discs, Becton, Dickinson and Company, Loveton, USA) and disc potencies (μg) were used: amikacin (30), amoxicillin + clavulanic acid (20/10), ampicillin (10), cefoxitin (30), ceftriaxone (30), cephalothin (30), chloramphenicol (30), ciprofloxacin (5), gentamicin (10), kanamycin (30), nalidixic acid (30), neomycin (30), nitrofurantoin (100), streptomycin (10), sulfisoxazole (1000), sulfamethoxazole + trimethoprim (23.75/1.25), trimethoprim (5) and tetracycline (30). The interpretation of the categories of susceptible, intermediate or resistant was based on the CLSI guidelines [36] and for the purpose of analysis, all readings classified as intermediate were considered as resistant where necessary. Reference strain of *Escherichia coli* ATCC 25922 was used as a quality control.

### Ethical consideration

The study protocol was ethically approved by the Institutional Review Board of College of Health Sciences, Addis Ababa University and National Research Ethics Review Committee (Permit#3-10/474/05 dated 29–03–2015). Individual oral informed consent was obtained from all adult participants and the parents or guardians of all children participated in the study.

### Statistical analysis

Data was analyzed using STATA software version 11. Prevalence of *Salmonella* was calculated as a percentage of *Salmonella* culture-positive stool samples among the total number of samples examined. Associations of putative risk factors as well as occurrence of protozoan pathogens with *Salmonella* infection in patients from health centers were assessed using both univariable and multivariable logistic regression, accounting for clustering of patients at the health center level by using cluster-robust standard errors. The variables used in multivariable logistic regression were sex, age, consumption of raw (meat, milk, vegetables), stool consistency, vomiting, and infection status with *Entamoeba histolytica* and *Giardia lamblia.* Results were reported statistically significant whenever the *p*-value was less than 5 %.

## Results

### *Salmonella* prevalence and association with patient feeding habits and other factors

All *Salmonella* isolates recovered from a single patient from both RVB and TTB, though in some cases (11.9 %), differed in antimicrobial resistance pattern; they all belonged to a single serotype. Therefore, only a single serotype per patient was considered for risk factor analysis and serotype distribution analysis, while all isolates (*n =* 67) were considered for antimicrobial resistance analysis. Seven hundred and sixty-five diarrheic patients from health centers (329 male and 436 female) and 192 patients (101 male and 89 female) from TASH were included in the study. Fifty-five (7.2 %) and 4 (2.1 %) patients from health centers and TASH were culture positive for *Salmonella*, respectively. The overall prevalence of *Salmonella* in the current study was 6.2 %. There was no difference in prevalence of *Salmonella* between male and female patients as well as among different age groups (Tables 1 and 2). Among eating habits of patients, 10.8 % of those consuming raw vegetables were *Salmonella* culture positive, whereas only 6.1 % of patients with no habit of eating raw vegetables were positive for *Salmonella.* Multivariable logistic regression analysis after adjusting for all pre-specified variables revealed statistically significant association of *Salmonella* infection status with consumption of raw vegetables (OR = 1.91, 95 % CI = 1.29–2.83, χ^2^ = 4.74, *p =* 0.025) (Table 2).

**Table 1:** Frequency of *Salmonella* infection among diarrheic out-patients attending health centers in Addis Ababa with respect to selected factors

Factor	Total	No (%) *Salmonella* positive	*P*-value
Sex			
Male	329	24(7.3)	0.92
Female	436	31(7.1)	
Consumption of raw meat			
No	622	44(7.1)	0.80
Yes	143	11(7.7)	
Consumption of raw milk			
No	720	51(7.1)	0.19
Yes	45	4(8.9)	
Consumption of raw vegetables			
No	571	34(6.0)	0.025
Yes	194	21(10.8)	
Vomiting			
No	714	51(7.1)	0.85
Yes	51	4(7.8)	
Age			
0–5 years	160	10(6.3)	
6–18 years	171	12(7.0)	0.77
19–45	359	27(7.5)	
>45	75	6(8)	
Stool consistency			
Mucoid	283	10(3.5)	
Loose	117	9(7.7)	0.005
Watery	365	36(9.9)	
*Entamoeba histolytica* infection			
Positive	145	14(9.7)	0.058
Negative	620	41(6.6)	
*Giardia lamblia* infection			
Positive	62	3(4.8)	0.46
Negative	703	52(7.4)

**Table 2:** Association of selected risk factors with *Salmonella* infection in diarrheic out-patients in Addis Ababa health centers

Variable	Crude odds Ratio and 95 % confidence interval		Adjusted odds Ratio and 95 % confidence interval	
Sex				
Male	1.03	0.61–1.74	1.02	0.57–1.85
Female				
Consumption of raw meat				
No	1.1	0.58–2.08	0.86	0.55–1.36
Yes				
Consumption of raw milk				
No	1.28	0.53–3.01	1.29	0.51–3.26
Yes				
Consumption of raw vegetable				
No	1.92	1.36–2.70	1.91	1.29–2.83
Yes				
Vomiting				
No	1.11	0.37–3.32	0.93	0.34–2.52
Yes				
Age				
0–5 years				
6–18 years	1.14	0.41–3.13	1.03	0.38–2.81
19–45	1.22	0.53–2.81	1.02	0.44–2.35
>45	1.32	0.32–5.57	1.27	0.33–4.93
Stool consistency				
Mucoid				
Loose	2.28	0.90–5.76	2.33	0.91–5.97
Watery	2.99	1.23–7.27	3.05	1.22–7.60
*Entamoeba histolytica* infection				
Positive	1.85	0.84–4.08	1.77	0.85–3.67
Negative				
*Giardia lamblia* infection				
Positive	0.64	0.25–1.60	0.64	0.26–1.56
Negative				

**Fig. 1:**
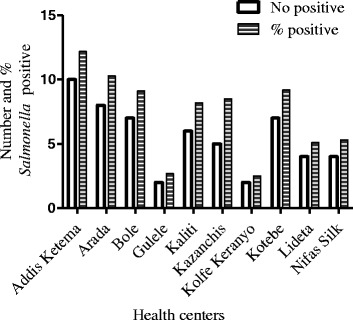
Prevalence of *Salmonella* among out-patients attending primary health centers in Addis Ababa

Although there was variability in the detection rate of *Salmonella* from patients attending different health centers, the difference was not statistically significant (*p =* 0.29) (Fig. 1). Having watery diarrhea (9.8 %) was significantly associated with *Salmonella* recovery compared to mucoid (7.7 %) and loose stool (3.5 %) using multivariable logistic regression analysis after adjusting for the other variables (OR = 3.3, 95 % CI = 1.23–8.88, χ^2^ = 10.54, *p =* 0.005). Comparison of patients with symptoms of vomiting to those with no vomiting revealed no significant difference in *Salmonella* culture-positivity (Tables 1 and 2).

### Prevalence of ova and parasites and concomitant infection with *Salmonella*

The most common pathogens detected were *Entamoeba histolytica* (19 %), *Giardia lamblia* (8.1 %), egg of *Hymenolepis nanna* (0.9 %) and egg/larvae of *Strongyloides stercoralis* (0.7 %) among patients from health centers, whereas only *E. histolytica* (0.5 %), *G. lambia* (4.3 %), and egg of *Tanea* species (1.1 %) were detected in patients from TASH (Table 3). Although *Salmonella* was commonly detected in patients positive for *E. histolytica* (9.7 %) compared to negative ones (6.6 %) *Salmonella* infection was not significantly associated with any of the parasites (Table 2).

**Table 3:** Common pathogens detected from stool samples of the study participants

	Health Centers		TASH	
Pathogen infection status	Number examined	Number (%) positive	^a^Number examined	Number (%) positive
*Entamoeba histolytica*	765	145(19)	188	1(0.5)
*Giardia lamblia*	765	62(8.1)	188	8(4.3)
*Hymenolepis nanna*	765	7(0.9)	188	0(0)
Strongloid worm/eggs	765	5(0.7)	188	0(0)
Hook worm	765	3(0.4)	188	0(0)
Ascaris egg	765	2(0.3)	188	0(0)
Eggs of Tanea	765	0(0)	188	2(1.1)

^a^Only 188 of 192 samples at TASH were examined for ova and parasites

### *Salmonella* Serotype distribution

Overall, 11 different serotypes were recovered, of which the majority were *S*. Typhimurium (22, 37.3 %) followed by *S.* Virchow (20, 33.9 %) and *S*. Kottbus (6, 10.2 %). Other serotypes such as *S.* Miami (*n =* 2), *S.* Kentucky (*n =* 2), *S.* Newport (*n =* 2), *S.* Enteritidis (*n =* 1), *S.* Braenderup (*n =* 1), *S.* Saintpaul (*n =* 1), *S.* Concord (*n =* 1), and *S.* V: ROUGH-O; − :- (*n =* 1) were also identified (Table 4).

**Table 4:** Relative proportion of *Salmonella* serotypes isolated from diarrheic patients in Addis Ababa and their resistance profile

Serotype	No.	An	Amp	Amc	C	Cro	Cf	Cip	Fox	Gm	K	Sxt	Tmp	Te	Su	S	Nitro	Na	N
Braenderup	1	0	0	0	0	0	0	0	0	0	0	0	0	0	1	1	0	0	0
Concord	1	0	1	1	1	1	1	0	1	1	1	1	1	0	1	1	1	0	0
Enteritidis	1	0	0	0	0	0	0	0	0	0	1	0	0	0	0	1	1	1	1
Kentucky	2	0	2	2	0	0	2	2	1	2	2	0	0	2	2	2	0	2	1
Kottbus	7	0	0	0	0	0	0	1	0	1	3	0	0	1	4	7	2	1	2
Miami	3	0	0	0	0	0	0	0	0	0	1	0	0	0	0	3	2	0	0
Newport	2	0	0	0	0	0	0	0	0	0	1	1	1	0	0	1	0	1	0
Sainpaul	1	0	0	0	0	0	1	0	0	0	1	0	0	0	1	1	0	0	1
Typhimurium	27	0	6	4	0	1	6	0	0	0	6	1	1	3	13	18	9	1	1
V:ROUGH-O;-:-	1	0	1	0	0	0	1	0	0	0	1	0	0	0	0	1	1	0	0
Virchow	21	0	1	1	1	0	1	0	0	1	6	0	0	3	4	14	11	10	3
Total	67	0	11	8	2	2	12	3	2	5	23	3	3	9	26	50	27	16	9
(%)	100	0	16.4	11.9	3	3	17.9	4.5	3	7.5	34.3	4.5	4.5	13.4	38.9	74.6	40.3	23.9	13.4

*An* amikacin, *Amp* ampicillin, *Amc* amoxicillin and clavulanic acid, *Cf* cephalothin, *C*, chloramphenicol, *Cro* ceftriaxone, *Cip* ciprofloxacin, *Gm* gentamicin, *K* kanamycin, *Tmp* trimethoprim, *Sxt* sulfamethoxazole + trimethoprim, *Te* tetracycline, *Su*-sulfisoxazole, *Nitro* nitrofurantoin, *Na*- nalidixic acid, *N* neomycin

### Antimicrobial Susceptibility

At least one isolate was resistant to all tested antimicrobial agents except amikacin to which all isolates were susceptible. Fifty (74.6 %), 27 (40.3 %), 26 (38.8 %), 23 (34.3 %), 12 (17.9 %), and 11 (16.4 %) of the isolates were resistant to streptomycin, nitrofurantoin, sulfisoxazole, kanamycin, cefalothin, and ampicillin, respectively (Table 4). Nine (13.4 %) of the isolates were pan-susceptible to 18 antimicrobials tested. Six of these isolates were *S*. Typhimurium, while the remaining 3 were *S.* Virchow. Resistance to 2 or more antimicrobials was recorded in 64.2 % of the isolates, while resistance to 3 or more antimicrobials was detected in 40.3 % of the total isolates. MDR to 5 or more antimicrobials was detected in 17 (25.4 %) of the isolates. Two strains of *S*. Kentucky isolated from patients in two different health centers were resistant to 11 antimicrobials. Likewise, one *S*. Typhimurium phagetype 193 and one *S.* Concord recovered from two diarrheic children at TASH were resistant to 12 and 13 antimicrobials tested, respectively. In general, 36 different resistance patterns (R-Pattern) were detected among the 67 isolates examined which is an indication of phenotypic diversity of *Salmonella* strains circulating in the study area. The dominant R-pattern was resistance to a single antimicrobial agent: streptomycin (*n =* 11) followed by resistance to both streptomycin and nitrofurantoin (6 isolates) (Additional file 1: Table S1).

### Association between serotype and antimicrobial resistance

Different serotypes appeared to exhibit differential resistance to some of the antimicrobials tested. For instance, resistance to ampicillin was noted in strains belonging to *S.* Concord, *S.* Kentucky, *S.* Typhimurium, *S.* V: ROUGH-O;-: and *S.* Virchow; while *S.* Kentucky and *S*. Kottbus were the only serotypes resistant to ciprofloxacin. In other cases, resistance to streptomycin was common among the serotypes (Table 4).

## Discussion

In the current study, 7.2 % of patients from the health centers and 2.1 % from TASH were culture positive for *Salmonella* species. The possible explanation for low prevalence of *Salmonella* in patients at TASH could be due to health center treated patient referral to this hospital or because not all of the stool samples collected at TASH were from diarrheic patients. The observed heterogeneity of *Salmonella* infection across health centers goes with the known hygienic levels of residential areas served by these health centers. Addis Ketema and Arada health centers are located at the center of the old Addis Ababa with larger human population and lots of slum areas with poor hygiene compared to Kolfe Keranyo and Gulele health centers, which are located at periphery of the city with relatively better hygienic condition.

The overall prevalence of *Salmonella* (6.2 %) in the current study is in agreement with previous studies in Addis Ababa (5.3 %) [20] and Jimma hospitals (6.2 %) [37] in pediatric patients. Previous studies in Addis Ababa in adult diarrheic out-patients showed a prevalence of 4.5 % [24] and 6.4 % [25]. Systematic review and meta-analysis of published works in Ethiopia over the period of 38 years has also shown a prevalence of 8.7 % in diarrheic children and 5.7 % in diarrheic adults [38].

*Salmonella* detection was more common in patients who consume raw vegetables. This suggests that raw vegetables could be one of the major vehicles for *Salmonella* infection in Addis Ababa. The common vegetables consumed by these patients were lettuce, tomatoes and green peppers. An accumulating body of evidence indicates that vegetables sold in markets could possibly be contaminated with *Salmonella* species*.* Indeed, studies conducted in Addis Ababa [39], Jimma (South–west Ethiopia) [40] and Mexico [41] showed that an appreciable percentage of vegetables sold in the respective markets are contaminated with *Salmonella* species*.* The use of fecally contaminated water for irrigation and washing of fresh produces can serve as a source of *Salmonella* contamination for raw vegetables in addition to cross-contamination in the kitchen [42]. More recently, *Salmonella* has been shown to colonize and be internalized into tomato during pre–and post–harvesting stages [43]. Special attention should, therefore, be paid to reducing the risk of infection of the public with *Salmonella* from raw vegetables. Appropriate pre–and post–harvest strategies of reducing contamination of vegetables by *Salmonella* and other enteric pathogens should be implemented.

The dominant pathogens other than *Salmonella* detected in the current study were *E. histolytica* followed by *G. lamblia.* However, no significant association of co-occurrence of parasites with *Salmonella* infection was detected.

*S*. Typhimurium followed by *S.* Virchow and *S.* Kottubus were the dominant serotypes in the current study unlike the reports by Beyene et al. [20] and Gebre-Yohannes et al. [44] where the dominant serotypes among NTS isolates were *S.* Concord followed by *S.* Typhimurium. In the present study, only one *S.* Concord was isolated from a one year old child at TASH. The difference between the studies could be attributed to differences in patient demography, season and place of collection. Furthermore, differences in dynamics of the sources of infection might have contributed to dominance of different serotypes in the respective studies. A previous study has shown that the epidemiology of NTS is characterized by the temporal dominance of certain successful clones followed by a decline and replacement with another clone [45]. The dominance of *S.* Typhimurium in the current study is in agreement with studies conducted in other sub-Saharan African countries including Kenya [46] and Congo [47].

Overall, rate of antimicrobial resistance is low in the current study compared to previous investigations. For instance Beyene et al. [20] reported 82.3 % resistance to ampicillin and 78.2 % to ceftriaxone, which is much higher than the current finding of 16.4 % and 3 %, respectively. Only 3 %, 7.5 %, 13.4 %, and 4.5 % of the isolates were resistant to chloramphenicol, gentamicin, tetracycline, and sulfamethoxazole + trimethoprim, respectively in the current study, while 81.4 %, 74.3 %, 39.8 % and 80.5 % of the isolates were resistant to the corresponding antibiotics, respectively, in the previously mentioned study. A separate study reported 59.7 %, 32.3 %, 61.7 %, 29 %, and 51.6 % resistance to ampicillin, cephalothin, chloramphenicol, tetracycline and Sxt, respectively in NTS isolates recovered from hospitalized patients at TASH in Addis Ababa [48]. The reason behind this discrepancy could be differences in serotype composition reported by the studies. As mentioned, the dominant serotype in the study by Beyene *et al.* [20] was *S*. Concord. However, only one isolate of this serotype was recovered in the current study. Though *S.* Concord was resistant to many antimicrobials in both studies, its rarity in the present study might have contributed to the relatively low levels of overall antimicrobial resistance that was observed. Furthermore, the previous studies were conducted in referral hospitals, where patients are admitted after being treated in health centers with various antimicrobials. Thus, patients could have been infected by clonal epidemic MDR strains of *S.* Concord or other serotypes. Some of the infections in the previous reports also might have been acquired from hospitals by MDR strains. Majority of the isolates in the current study were, however, collected from patients at primary health centers, with minimized prior exposure to antimicrobials. The fact that 2 of the 4 isolates from TSRH in the current study were resistant to 11 and 13 antimicrobials supports this assertion. Similar decrease in prevalence of resistance in NTS in rural district hospital has also been reported from Kenya [49].

Although we have no data on the antimicrobial use for diarrheic patients in the current study, the recent standard treatment guideline prepared by Drug Administration and Control Authority of Ethiopia for severe cases of infectious gastroenteritis including NTS recommends sulfamethoxazole + trimethoprim, ciprofloxacin and chloramphenicol [50]. However, resistance to these antimicrobials was low in *Salmonella* isolates in the current study probably due to the fact that these isolates were mainly obtained from patients attending primary health centers and were not exposed to prolonged selective pressure of antimicrobials. Drugs like streptomycin, nitrofurantoin, sulfonamides and ampicillin have long been used for management of various infections in the country and high rate of resistance to these drugs might have developed as a consequence of this prolonged use.

## Conclusions

There was no significant association of *Salmonella* infection with co-occurring parasites but there was significant association of *Salmonella* infection with consumption of raw vegetables. The dominant NTS serotypes at the primary health care units in Addis Ababa were *S.* Typhimurium, *S*. Virchow and *S*. Kottbus with variable antimicrobial resistance phenotypes. Although their proportion was low, *S.* Kentucky and *S.* Concord demonstrated extensive MDR. Further characterization on molecular resistance determinants and continuous monitoring of circulating serotypes and antimicrobial resistance profile is recommended. As the extent of MDR appears to be dependent on serotypes involved, it is vital to have at least one national *Salmonella* reference laboratory to conduct serotyping of *Salmonella* isolates from all regions of Ethiopia.

## Additional file

Additional file 1: Table S1. Resistance pattern of *Salmonella* serotypes recovered from the study participants. (DOCX 15 kb)

## Abbreviations

ALIPB: Aklilu Lemma Institute of Pathobiology
MDR: Multi-drug resistance
NTS: Non-typhoidal *Salmonella*
RVB: Rappaport-Vassiliadis Broth
TASH: Tikur Anbessa Specialized Hospital
TTB: Tetrathionate broth
WHO: World Health Organization
