# Supplementary material for: Non-typhoidal Salmonella serotypes, antimicrobial resistance and co-infection with parasites among patients with diarrhea and other gastrointestinal complaints in Addis Ababa, Ethiopia
Source: BMC Infect Dis. 2015 Nov 4;15:497. doi: 10.1186/s12879-015-1235-y (PMC4634906; doi:10.1186/s12879-015-1235-y)
Supplement: Additional file 1: Table S1. — Resistance pattern of Salmonella serotypes recovered from the study participants. (DOCX 15 kb) [file 12879_2015_1235_MOESM1_ESM.docx]

Aditional file 1: Table S1. Resistance pattern of *Salmonella* serotypes recovered from the study participants

| R-pattern | No. of antimicrobial agents | No of isolates | Serotypes involved (No.) |
| --- | --- | --- | --- |
| Sensitive | 0 | 9 | Typhimurium PT-1(2), Typhimurium PT-2(2), Typhimurium PT-193(2), Virchow(3) |
| S | 1 | 11 | Kottbus(1),Miami(1),Newport(1),Typhimurium PT-1(1), Typhimurium PT-1(1), Typhimurium PT-126(2),Virchow(4) |
| Su | 1 | 1 | Typhimurium PT-126(1) |
| Nitro | 1 | 2 | Virchow(2) |
| K | 1 | 1 | Virchow |
| SNa | 2 | 1 | Typhimurium PT-126(1), |
| SNitro | 2 | 6 | Typhimurium PT-126(1) Typhimurium PT-1(1), (2),Birchow(2)Kottbus(1),Maimi(1) |
| SuS | 2 | 5 | Braenderup(1),Kottbus(1),Typhimurium -AT(1), Typhimurium-PT-2(1), Typhimurium-PT-126 |
| KNitro | 2 | 1 | Virchow |
| KS | 2 | 2 | Entertidis -AT(1), Typhimurium –PT 126(1) |
| GmS | 2 | 1 | Virchow |
| KSuS | 3 | 1 | Typhimurium PT-126 |
| KSNitro | 3 | 1 | Miami(1) |
| KSN | 3 | 1 | Kottbus(1) |
| TeSNitro | 3 | 1 | Virchow |
| SuSNitroN | 4 | 1 | Virchow |
| TeSuSNitro | 4 | 2 | Kottbus(1),Typhimurium-PT-126(1) |
| KSNitroNa | 4 | 1 | Virchow |
| KSuSN | 4 | 1 | Kottbus |
| KSxtTmpNa | 4 | 1 | Newport |
| SuSNitroNaN | 5 | 1 | Virchow |
| CfKSuSN | 5 | 1 | Saintpaul |
| CfKSuSNitro | 5 | 1 | Typhimurium-PT-1 |
| AmpTeSuSNitro | 5 | 1 | Typhimurium-PT-66 |
| AmpCfKSNitro | 5 | 1 | V:ROUGH-O;-:- |
| KSuSNitroN | 5 | 1 | Virchow |
| KTeSuSNitro | 5 | 1 | Virchow |
| CipGmKSuSNitro | 6 | 1 | Kottbus |
| AmpAmcCfKSuNitro | 6 | 1 | Typhimurium-PT-3 |
| AmpAmcCCfTeS | 6 | 1 | Vircchow |
| Amp CfKSuSNitro | 6 | 1 | Typhimurium-PT-3 |
| AmpAmcCfKTeSuNitro | 7 | 1 | Typhimurium- PT-3 |
| AmpAmcCfKSuSNitro | 7 | 1 | Typhimurium-PT-3 |
| AmpAmcCfCipGmKTeSuSNaN | 11 | 1 | Kentucky |
| AmpAmcCfCipFoxGmKTeSuSNa | 11 | 1 | Kentucky |
| AmpAmcCroCf,KSxtTmpTeSuSNitroN | 12 | 1 | Typhimurium-PT-193 |
| AmpAmcCCroCfFoxGmKSxt TmpSuSNitro | 13 | 1 | Concord |

PT-Phage type, AT-Atypical; An-amikacin, Amp,ampicillin; Amc, amoxicillin and clavulanic acid; Cf, cephalothin; Cip, ciprofloxacin; C-chloroamphenicol, Fox, cefoxitin, Cro-ceftriaxone Gm, gentamicin; K, kanamycin; Tmp, trimethoprim; Sxt, sulfamethoxazole + trimethoprim, Te, tetracycline, Su-sulfisoxazole; S-Streptomycin; Nitro, nitrofurantoin; Na-nalidixic acid, N, neomycin
